# Reducing maternal and child oral health disparities in Sub-Saharan Africa through a community-based strategy

**DOI:** 10.3389/froh.2024.1429332

**Published:** 2024-06-28

**Authors:** Abiola Adeniyi, Gladys Akama, Ochiba Lukandu, Justus E. Ikemeri, Anjellah Jumah, Sheilah Chelagat, Anusu Kasuya, Laura Ruhl, Julia Songok, Astrid Christoffersen-Deb

**Affiliations:** ^1^ School of Policy and Global Studies, Fairleigh Dickinson University, Vancouver, BC, Canada; ^2^ Department of Community, Preventive Dentistry and Periodontology, School of Dentistry, Moi University College of Health Sciences, Eldoret, Kenya; ^3^ Department of Maxillofacial Surgery, Oral Medicine, Oral Pathology and Radiology, School of Dentistry, Moi University College of Health Sciences, Eldoret, Kenya; ^4^ Population Health, Academic Model Providing Access to Healthcare, Eldoret, Kenya; ^5^ Department of Medicine, Indiana University Medical School, Bloomington, IN, United States; ^6^ Child Health and Pediatrics, Moi University College of Health Sciences, Eldoret, Kenya; ^7^ Department of Obstetrics and Gynecology, University of Toronto, Toronto, ON, Canada

**Keywords:** maternal and child health, oral health disparities, community-based strategy, preventive oral healthcare, Sub-Saharan Africa

## Abstract

Oral conditions disproportionately affect mothers and children in Sub-Saharan Africa, due to biological vulnerabilities, a scarcity of oral health workers, deficient preventive strategies, and gender-based barriers to care. The World Health Organization (WHO) recommends integrating oral health into broader health delivery models, to reduce these disparities. We propose integrating preventive oral healthcare into community-based programs to bridge these gaps. We examine integrating preventive oral healthcare into Western Kenya's Chamas for Change (*Chamas*) community-based program which aims to reduce maternal and child health disparities. *Chamas* incorporates women's health and microfinance programs best practices to produce a low-cost, community-driven, sustainable, and culturally acceptable health delivery platform. Our strategy is based on the Maternal and Child Oral Health Framework and uses the WHO Basic Package of Oral Care principles. This framework prioritizes community involvement, cultural sensitivity, regular screenings, and seamless integration into general health sessions. We discuss the strengths, weaknesses, opportunities, and threats to enriching *Chamas* with oral health promotion activities. It is crucial to assess the effectiveness, sustainability, and acceptability of the proposed strategy through implementation and evaluation. Future studies should investigate the long-term impact of integrated oral health models on community health and oral health disparity reduction in Africa.

## 1 Introduction

The World Health Organization (WHO) reports that 3.5 billion people, roughly half of the global population, suffer from oral diseases such as dental caries, periodontal disease, edentulism, and oral cancer (1–3). These conditions share common risk factors and social determinants with many noncommunicable diseases (NCDs) (2–5). They also disproportionately affect vulnerable people, in particular those of low socio-economic status and rural residents (6, 7). Women and children face additional barriers due to gender-based inequalities (8, 9). Oral health has an extremely important role in general well-being and quality of life, particularly for women and children. Moreover, oral diseases despite being largely preventable, still place a considerable financial strain on households (9–12). This situation highlights the need for innovative and cost-effective strategies to address the inequitable impact of oral diseases on mothers and children (2, 3).

In 2019, oral diseases affected over 480 million people —44% of the population -, in Sub-Saharan Africa including Kenya, marking a significant increase over the last three decades (7, 13). This rise is largely due to structural determinants such as financial barriers, lack of oral health policies, inadequate oral health infrastructure and insufficient healthcare providers awareness, which collectively limit access to oral health care (7, 14, 15). These challenges are worse for women and girls, who face additional cultural and economic barriers to health services, including oral health care (16–18). With 25% of the African population suffering from untreated dental caries, there is an urgent need for interventions designed to address these barriers and improve oral health outcomes (6, 13).

Our article aims to bridge the gap in access to oral health care in Sub-Saharan Africa by proposing the incorporation of oral health activities into community-based programs targeting mothers and children in the region. Using the Chamas for Change program (*Chamas*) in Western Kenya as a prototype, we advocate for the integration of preventive oral health care into community-based interventions. We describe how this can be implemented to address individual and community oral health disparities while aligning with global oral health goals and the Kenyan national oral health strategic plan (19–23). We include a Strengths, Weaknesses, Opportunities, and Threats (SWOT) analysis of this solution to illustrate the rationale and benefits. We posit that our proposal will improve oral health outcomes and overall well-being in underserved populations. Ultimately, we emphasize the essential role of oral health within the broader public health context, and advocate for its recognition as a vital element of comprehensive well-being (22).

### 1.1 The *Chamas* program as a suitable community-based program

The *Chamas* program was launched in 2012 by the Academic Model Providing Access to Healthcare (AMPATH) in collaboration with the Government of Kenya. This community-based program focuses on addressing the inequities that drive maternal and infant mortality in Kenya by creating a community-based service delivery platform that facilitates access to care, financial empowerment, peer support and sexual and reproductive health (SRH) advocacy (24). Using the longstanding tradition of community-based support structures in East Africa, “*Chamas*,” which means “groups with purpose” in Swahili, addresses the health, social, and financial needs of pregnant women and mothers in the first 3 years postnatally.

*Chamas* creates an affordable, community-run, self-sustaining, and culturally acceptable integrated service delivery platform by integrating best practices from microfinance and women's health programs. *Chamas*' 3-year mentored program improves perinatal outcomes through health, social, and financial literacy (24–26). The program is facilitated by Community Health Promoters (CHPs), that are community nominated part-time government volunteers, under the supervision of Community Health Extension Workers (24). The CHPs undergo comprehensive training, including a 2-week session covering various health topics, with a focus on maternal neonatal and child health (MNCH). These trained individuals conduct routine health visits, collect health information, identify health problems, and refer individuals to health facilities when needed.

The *Chamas* program engages women in groups of 15–20 participants, meeting twice a month over a 12-month period to attend 24 CHP-facilitated sessions annually. These sessions cover a range of health and social topics, from antenatal care to intimate partner violence, utilizing illustrated flipcharts and participatory discussions. Members commit to practicing key MNCH behaviors and have the option to participate in the “Group Integrated Savings for Health and Empowerment” (GISHE) a table-banking program. This optional program enables members to contribute to a microfinance scheme, fostering financial empowerment. *Chamas* has been successfully integrated into the primary care delivery framework across counties in Western Kenya. Currently operational in 5 counties (Trans Nzoia, Busia, Bungoma, Uasin-Gishu and Elgeyo Marakwet) and 15 sub-counties, the program has 430 groups with 4,701 participants.

The *Chamas* program addresses different stages of maternal and child well-being over 3 years. Prenatal and early postnatal care, family planning, exclusive breastfeeding, and financial literacy are covered in year 1 for pregnant women in their first or second trimesters. In year 2, which covers the first year postpartum, the programme emphasises early parenting, childhood immunizations, complementary feeding, and bank account opening. Year 3, for the second-year postpartum, emphasises positive parenting, helps participants apply for government loans, and encourages small businesses. Overall, the program has demonstrated success, with positive effects on key health outcomes, fostering participant's empowerment, and community building (24–26).

*Chamas* women have demonstrated significantly higher odds of achieving positive MNCH outcomes, including delivering in a healthcare facility under the supervision of a trained healthcare provider, receiving a 48-h postpartum visit from a CHP, exclusively breastfeeding for at least 6 months, and adopting a postpartum contraceptive method (24, 25, 27). *Chamas* participation was associated with five times the odds of facility delivery and a 5-fold increase in the likelihood of a CHP conducting a post-delivery home visit (24). Moreover, participants reported greater infant immunization completion rates, reduced parental stress, and fewer reports of abuse (24–26). Beyond health impacts, the program promotes community-centered care, builds resilience during pandemics and other health emergencies, fosters increased peer support, enhances women's empowerment, strengthens family and community support, and leads to increased uptake of National Health Insurance.

While the *Chamas* program effectively addresses various MNCH concerns, its influence on oral health outcomes remains unexamined. Nonetheless, given its demonstrated success in enhancing overall maternal and child health outcomes and promoting community empowerment, integrating preventive oral health activities into the *Chamas* framework offers a promising avenue for addressing oral health disparities among mothers and children in Kenya. It also holds promise for delivering preventive oral health to people living in rural areas and those who experience limited access to care. Considering the *Chamas* program's significant impact, the interrelation between oral health and systemic well-being, and the imperative for oral health interventions for mothers and children in Kenya, we propose the integration of preventive oral health activities within the *Chamas* activities framework.

### 1.2 Proposed strategy for integrating oral health promotion activities into the Chamas program

Our proposed solution aims to integrate oral health education and screening sessions into the 3-year *Chamas* program. We plan to integrate comprehensive oral health education both as standalone sessions and within selected existing health and social education sessions. Our proposal is to feature 24 oral health promotion topics in the first 2 years of the *Chamas* program, with a detailed breakdown provided in Table 1. These include four standalone topics and the inclusion of oral health content in nine existing sessions in the first year. This is followed by one standalone topic and the integration of oral health information into ten existing sessions in the second year. Drawing upon the WHO training curriculum, we plan to equip CHPs with the necessary knowledge to impart basic oral health information (28). We intend to train the CHPs via a 3-day workshop facilitated by a trained dental public health specialist. We will supplement the initial training with annual refreshers that align with current MNCH training. This equips CHPs with WHO-guided knowledge to deliver community-based oral health interventions effectively.

**Table 1:** Proposed revised *Chamas* curriculum including oral health education topics.

Lesson	health topic	Social topic	Suggested oral health topic
Year 1 curriculum			
1	Importance of women attending ANC clinics	Upholding the goals of the Chama	Importance of oral health for mother and child^b^
2	Danger signs during pregnancy and after delivery	Table banking (Savings and loans)	
3	Physical exercises during pregnancy	National Health Insurance Fund (NHIF) Super Cover	Common symptoms and signs of dental disease^b^
4	Importance of facility delivery^a^	Budget planning	Importance of regular dental visits for mother and child
5	Preventing mother-child transmission: planned pregnancy, early ANC, ART, delivery in facility, infant follow-up to 18 months^a^	Disclosing HIV status to your family	Demystifying myths about oral health in pregnancy and childhood
6	Pregnancy specific complications: GMD, Pre-eclampsia/eclampsia, and Anemia	Good nutrition during pregnancy^a^	Healthy diet for oral health
7	Pre-term labor and labor complications	Mutual sexual satisfaction between a man and a woman	
8	Negative pregnancy outcomes: miscarriage, stillbirth, and abortion^a^	Male involvement during pregnancy and infancy	Complications of untreated dental disease
9	Danger signs after delivery for mama: fistula, PPH, and other red flags	Supporting the birth of a child in your Chama	
10	Danger signs in your newborn: 4 h up to 2 weeks	Community advocacy for our health	Prevention of early childhood caries^b^
11	Exclusive breastfeeding^a^	Adolescent pregnancy and school dropouts	Breastfeeding effects on the teeth
12	Importance of Kangaroo care between mother and child	Single parenting or losing a spouse	
13	Teratogens: alcohol, tobacco, and other drug use^a^	Domestic violence	How baby's teeth develop
14	Congenital anomalies or birth defects^a^	After delivery welfare up to 1 year	Neonatal teeth
15	Importance of women attending PNC clinics	Awareness of the child's well-being	
16	Postpartum depression	Promoting a good relationship with your husband in the home	
17	Infant and childhood immunizations^a^	Promoting a good relationship with mother-in-law and sister-in-law	Screening for dental disease
18	SIDS and safe sleep	Setting routines for the infant: sleeping	
19	Infant growth monitoring^a^	Setting routines for the infant: eating^a^	Milestones in dental development
20	Infant development^a^	Farming and rearing chicken	Preventing dental caries and gum disease^b^
21	Intro to family planning and birth spacing	Agribusiness	
22	Family planning: IUCD, tubal ligation, and vasectomy	Communication in relationships	
23	Family planning: Jadelle and implanon/nexplanon	Female empowerment: self esteem and body image^a^	
24	Family planning: pill, depo, and condoms	Conflict resolution within a Chama	
Year 2 curriculum			
25	Complementary feeding for your infant^a^	Cooking in clean air	Nutrition and your child's teeth
26	HIV testing in infants after birth	Reducing stigma towards members in the community with HIV	
27	STIs beyond HIV: chlamydia, syphilis, and gonorrhoea^a^	National Health Insurance Fund (NHIF) SupaCover	STI's and oral health
28	Diseases under surveillance in children: measles, polio, and pneumonia^a^	Avoiding road traffic accidents	Surveillance diseases and oral health
29	Childhood fever: causes and management^a^	Steps of childhood development	Febrile illness and your child's teeth
30	Diarrheal diseases^a^	Clean water	Teething management
31	Deworming and complications of worm infections	Nutrition and cleanliness^a^	Good oral hygiene
32	Malaria	Importance of play	
33	Health hazards and childproofing	Learning styles in your child	
34	Basic first aid: choking and burns^a^	Emergency preparedness and response	Simple dental pain home remedies
35	Advanced first aid	Violence in the community	Trauma to your child's tooth
36	Common poisons: organophosphates, kerosene, and paracetamol	Hygiene in the home	
37	Childhood skin diseases: scabies, jiggers, and atopic dermatitis	Role modeling for your child^a^	Role modeling oral hygiene
38	Tuberculosis	Showing love and building trust	
39	Chronic diseases: diabetes, cancer, and heart health^a^	Consistency and daily routines	Oral health and systemic health connection
40	Eliminating female genital mutilation	Gender-based violence	
41	Male circumcision	Child sexual abuse	
42	Cervical cancer screening: overcoming fears and misconceptions	Discipline and correction	
43	Breast cancer screening	Learning from daily activities	
44	Infertility: causes and support	Toilet training your Child	
45	Developmental disorders and disabilities	Supporting individuals in the community with disability	
46	Mental health disorders	Dealing with life stresses	
47	Dental health for your child^b^	Raising a teenage child	
48	Healthy lifestyles	Planning for the future: raising your child	

^a^ Existing sessions where relevant oral health information can be incorporated. Suggested topics for integrated sessions are included in italics.

^b^ Standalone oral health sessions.

Our strategy also includes conducting annual oral health screenings in the second and third years, based on the WHO's Basic Package of Oral Care (BPOC) (29). This package includes Oral Urgent Treatment (OUT), the use of Affordable Fluoride Toothpaste (AFT), and Atraumatic Restorative Treatment (ART). Community Oral Health Workers will oversee these screenings, facilitate access to dental care facilities and administer treatments such as AFT and ART where necessary. This approach seeks to enhance the *Chamas* program by embedding preventive oral healthcare within routine care, to reduce maternal and child oral health disparities.

**Figure 1:**
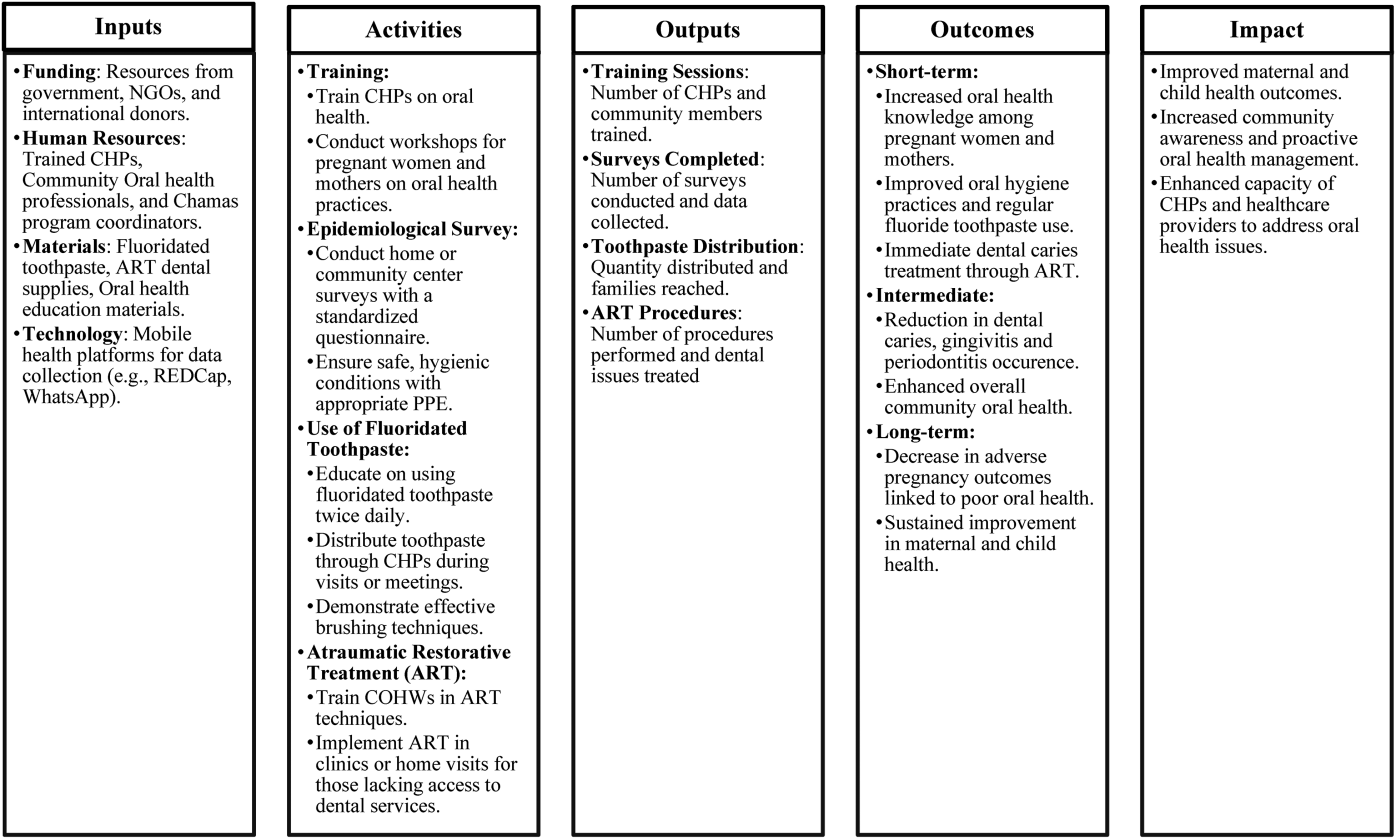
Logic model depicting the integration of oral health promotion activities within the Chamas program.

Our Logic Model (Figure 1) for integrating oral health promotion activities in the *Chamas* Program outlines a structured approach to improving maternal and child health through enhanced oral health practices. It includes inputs such as funding, trained human resources, necessary materials, and technology for data collection. Key activities involve training CHPs, conducting epidemiological surveys, promoting the use of fluoridated toothpaste, and providing ART procedures. Outputs will measure the reach and effectiveness of these activities, including the number of training sessions, surveys completed, toothpaste distributed, and ART procedures performed. Outcomes are categorized into short-term, intermediate, and long-term effects, highlighting improvements in oral health knowledge, practices, and overall health outcomes. The ultimate impact aims to enhance maternal and child health, increase community awareness, and build the capacity of healthcare providers to address oral health issues effectively.

## 2 Discussion

The Chamas program is an innovative, integrated health strategy that addresses maternal and child oral health disparities in Sub-Saharan Africa. In this section, we use a SWOT analysis (Table 2) to emphasize the strengths, weaknesses, opportunities, and threats of integrating preventive oral health activities into a community-based program-*Chamas*— that has enhanced MNCH outcomes in rural Kenya (24–27).

**Table 2:** A summary of the SWOT analysis for integrating oral health into the *Chamas* program.

SWOT analysis component	Description
Strengths	• Chamas groups are well-established and trusted within communities, making implementation and uptake easier. • Utilizes the same venues, staff, and materials for maternal and oral health, reducing costs and increasing efficiency. • The program's alignment with community norms and values makes it an effective oral health promotion platform. • Emphasizes early detection and prevention to reduce long-term disease burden and integrate oral health with general healthcare during routine child wellness visits. • Financial Empowerment enables community members to access essential preventive products, potentially improving long-term health outcomes
Weaknesses	• Lack of adequate oral health facilities and professionals, which can limit service delivery. • Proper training of Chamas facilitators on oral health is necessary and could be resource intensive. • May complicate Chamas meetings and extend their duration, which could affect attendance and engagement. • There may be a lack of oral health expertise, requiring new hires or collaborations. • The patriarchal society structure demand creative and culturally sensitive strategies to improve effectiveness. • Additional funding is necessary for fluoride treatments, educational materials, and professional dental care referrals
Opportunities	• Educating antenatal care providers on oral health can significantly improve patient outcomes, reduce disease metrics, and increase service utilization. • Aligns with Kenya's increasing recognition of oral health in NCD, PHC and NCD policies. • Offers a model that can be scaled and replicated in other regions, enhancing oral health equity among underserved populations. • Potential for support and partnerships with dental associations, health NGOs, and government units, particularly for expanding health services and public health outreach. • Support from community leaders can substantially boost oral health's visibility and facilitate improvements across Africa
Threats	• Oral health may be deprioritized due to other pressing health challenges, risking necessary resource diversion. • The existing fee-for-service model and sectoral resistance pose substantial challenges. • Shifting cultural perceptions and ensuring affordability/accessibility of essential products like fluoride toothpaste require persistent advocacy. • Relies heavily on adequate funding, which can be uncertain and fluctuating. • Changes in health policy or funding priorities could jeopardize support for the integrated program

Our planned integration leverages our current infrastructure to expand service delivery. *Chamas*' established structure within communities and its high level of trust and engagement are therefore key strengths. It will be easy to achieve smooth implementation and higher uptake of the oral health intervention with these attributes. This will help reduce the long-term oral disease burden among vulnerable populations through our intervention, which focuses on prevention and early detection (29). *Chamas* a viable tool for oral health promotion because it allows the use of the same venues, staff, and materials for oral health making it cost-effective and efficient. The CHPs can deliver oral health education aligning with global and Kenyan policies to optimize resource use and health outreach (1, 3, 5, 15, 19, 22).

We also envisage integrating oral health into routine early child wellness visits and utilizing *Chama*s' peer-support systems to improve oral health at the individual, familial, and community levels (28, 30–32). This can foster a culture of prevention for oral health, improve access to oral healthcare through annual screenings, and integrate oral health with general healthcare (5, 14). In addition, our program's financial empowerment component can assist members in accessing essential preventive oral health products, such as fluoride toothpastes (33).

We have identified several weaknesses in the proposed integration. The oral health infrastructure in Kenya is insufficient, and there is a lack of awareness about the importance of oral health, including among non-dental health professionals (19, 34, 35). A strong public health system that includes oral health is needed for the oral health component to be effective. Kenya's free maternity services greatly contributed to the improvement of MNCH outcomes for *Chamas*, indicating a potential model for oral health. Creative solutions to address the cultural and socioeconomic challenges, especially the patriarchal nature of Kenyan society is important. Securing extra funding for fluoride treatments, educational materials, and potential professional oral healthcare referrals could pose a challenge. Training *Chamas* facilitators in preventive oral health may require significant resources, and potentially complicate *Chamas* meetings thus extending their duration. *Chamas* attendance may be impacted, and new skills or collaborations may be required, both of which can create logistical and budgetary challenges.

There are a lot of opportunities to make a positive impact by integrating preventive oral health within *Chamas*. Our proposal can greatly enhance patient outcomes by training community health providers on oral health. This will empower women to make informed health decisions for themselves and their children. This training can lead to reduce gingival disease metrics, improve plaque control, and increase utilization of oral health services (36, 37). This will significantly decrease oral health issues, improve children's nutrition and overall health, and prevent maternal infections through these clinical improvements.

Our proposed model is scalable, can improve oral health competency among non-dental providers, promote interprofessional collaboration and address the critical oral health workforce gap (12, 38, 39). Our proposal aligns with Kenya's health policies and recognizes the importance of oral health in managing NCDs, enhancing primary healthcare and supporting the achievement of universal health coverage (19, 35). Moreover, we can partner with dental associations, health NGOs, and secure government support to enhance public health outreach. This can enable us to replicate this model in other regions (10, 39).

However, there are a few potential threats that could undermine this integration. The initiative may lose necessary resources due to competing health priorities and the low prioritization of oral health in the health system. There is need for broad-based support from diverse stakeholders to ensure the sustainability of the program, which can be challenging in many African settings. The project can face significant hurdles due to resistance from the healthcare sector and the prevailing fee-for-service delivery model given the high poverty levels in rural communities. Inaccurate information about oral health may greatly impede the acceptance and effectiveness of the intervention. Challenges in educating and engaging the public due to recurring myths and misunderstandings surrounding oral health practices can also occur. Strong advocacy is needed to change cultural perceptions and ensure that essential products, such as fluoride toothpaste, are affordable and accessible.

Our proposal's stability and continuity may be threatened by fluctuations in funding and shifts in health policy or funding priorities. These changes may jeopardize support for the integrated program. Moreover, there is need to coordinate effectively among different health workers and sectors to ensure successful integration. This can be challenging, especially in settings with limited resources. There is also a risk of diluting the quality of maternal and child health services if the program becomes overly diversified or if resources are overly stretched. This dilution could compromise the effectiveness of both the existing services and the new oral health initiative, ultimately impacting the overall health outcomes of the communities served. Each of these threats needs careful consideration and strategic planning to ensure that the potential benefits of the program are not outweighed by these substantial challenges. By addressing these threats, the program can enhance its effectiveness and sustainability, thereby improving maternal and child health outcomes through integrated and comprehensive healthcare services.

## 3 Conclusion

Integrating preventive oral health activities into the *Chamas* program aligns with global and Kenyan health policies, making it essential for achieving global targets for integrated healthcare delivery and improving maternal and child oral health. The proposed integration promises substantial improvements in oral health equity and enhancing overall well-being in Kenyan communities. Despite challenges posed by societal structures and healthcare system constraints, strategic planning and robust advocacy can overcome these obstacles, paving the way for a healthier future for Kenya's rural populations. Through this integrated approach, community-based programs like *Chamas* can significantly elevate maternal and child health standards by incorporating essential oral health services.

## Data Availability

The original contributions presented in the study are included in the article/Supplementary Material, further inquiries can be directed to the corresponding author.
